# Draft Genome Sequence of *Xanthomonas arboricola* pv. pruni Strain Xap33, Causal Agent of Bacterial Spot Disease on Almond

**DOI:** 10.1128/genomeA.00440-14

**Published:** 2014-06-05

**Authors:** J. Garita-Cambronero, M. Sena-Vélez, A. Palacio-Bielsa, J. Cubero

**Affiliations:** a Departamento de Protección Vegetal, Laboratorio Bacteriología, Instituto Nacional de Investigación y Tecnología Agraria y Alimentaria (INIA), Madrid, Spain; b Centro de Investigación y Tecnología Agroalimentaria de Aragón (CITA), Zaragoza, Spain

## Abstract

We report the annotated genome sequence of *Xanthomonas arboricola* pv. pruni strain Xap33, isolated from almond leaves showing bacterial spot disease symptoms in Spain. The availability of this genome sequence will aid our understanding of the infection mechanism of this bacterium as well as its relationship to other species of the same genus.

## GENOME ANNOUNCEMENT

*Xanthomonas arboricola* pv. pruni (synonym *Xanthomonas campestris* pv. pruni) is a Gram-negative plant-pathogenic bacterium that causes bacterial spot disease of stone fruits and almond, one of the major threats of *Prunus* fruit crops. Symptoms occur on leaves, fruits, and twigs, ranging from necrotic angular lesions on leaves and sunken lesions on fruits to cankers on twigs. *X. arboricola* pv. pruni can be very damaging when severe infections occur on highly susceptible cultivars (1–4). This bacterium is considered a quarantine pathogen in the European Union phytosanitary legislation and in the European and Mediterranean Plant Protection Organization (EPPO) (5, 6). Here we report the draft genome sequence of *X. arboricola* pv. pruni strain Xap33 (CITA33), isolated from symptomatic almond leaves (*Prunus amygdalus* cv. Guara) cultivated in Aragón, Spain.

The genome of *X. arboricola* pv. pruni strain Xap33 was sequenced by the Ion Torrent Personal Genome Machine (PGM) (Life Technologies) instrument (579.53 Mb; 95-fold coverage). *De novo* assembly using CLC Genomics Workbench 7.0 (CLC bio, Denmark) produced a total length of 5,104,864 bp (G+C content, 65.43%) placed in 501 contigs with an *N*_50_ of 34,438 bp and a maximum contig length of 126,681 bp. The annotation for the Xap33 genome sequence was conducted using the automated Bacterial Annotation System (BASys) (7), the Rapid Annotation using Subsystem Technology server (RAST) (8), and the Prokaryotic Genome Annotation Pipeline (9). RNAmmer 1.2 and tRNAscan-SE 1.21 were used to predict rRNAs and tRNAs, respectively (10, 11).

The annotation for the Xap33 genome sequence detected 4,348 coding sequences, 3 rRNAs, and 50 tRNAs, representing 436 subsystems compared to 461 subsystems found in *Xanthomonas axonopodis* pv. citri strain 306, which is the most closely related bacterium based on nucleotide similarity in the RAST database. 16S rRNA comparison analysis performed with BLASTn (12) detected 99 to 100% identity to other *Xanthomonas* strains, including the *X. arboricola* pv. juglandis strain LMG 747. In addition, whole-genome nucleotide comparison using BLASTn against 214 complete and draft genomes from several *Xanthomonas* strains determined that the Xap33 genome sequence has 99% identity to the draft genome sequences of *Xanthomonas arboricola* pv. pruni strains MAFF301420, MAFF301427, and MAFF31562. Furthermore, in order to check the robustness of the Xap33 genome sequence, a multilocus sequence analysis using partial sequences of the housekeeping genes *dnaK*, *fyuA*, *gyrB*, and *rpoD* (13, 14) clustered the strain Xap33 in the *X. arboricola* pv. pruni phylogroup, revealing a high similarity (99.89%) to the type strain *X. arboricola* pv. pruni ICMP 51 (CFBP 2535). The genome information presented here will allow genetic analysis and manipulation of *X. arboricola* pv. pruni Xap33, as well as future comparative studies with other *Xanthomonas arboricola* pathovars. This new information will help in understanding the molecular mechanisms in the plant-pathogen interactions related to bacterial spot disease on almond.

### Nucleotide sequence accession numbers.

This whole-genome shotgun project has been deposited in DDBJ/EMBL/GenBank under the accession no. JHUQ00000000. The version described in this paper is version JHUQ01000000.
